# Associations of Serum Testosterone and SHBG With Incident Fractures in Middle-aged to Older Men

**DOI:** 10.1210/clinem/dgae703

**Published:** 2024-10-07

**Authors:** Louise Grahnemo, Ross J Marriott, Kevin Murray, Lauren T Tyack, Maria Nethander, Alvin M Matsumoto, Eric S Orwoll, Dirk Vanderschueren, Bu B Yeap, Claes Ohlsson

**Affiliations:** Department of Internal Medicine and Clinical Nutrition, Institute of Medicine, Sahlgrenska Osteoporosis Centre, Centre for Bone and Arthritis Research at the Sahlgrenska Academy, University of Gothenburg, Gothenburg SE-413 45, Sweden; School of Population and Global Health, University of Western Australia, Perth, Western Australia, Australia; School of Population and Global Health, University of Western Australia, Perth, Western Australia, Australia; Department of Endocrinology and Diabetes, Fiona Stanley Hospital, Perth, Western Australia, Australia; Department of Internal Medicine and Clinical Nutrition, Institute of Medicine, Sahlgrenska Osteoporosis Centre, Centre for Bone and Arthritis Research at the Sahlgrenska Academy, University of Gothenburg, Gothenburg SE-413 45, Sweden; Department of Medicine, University of Washington School of Medicine, and Geriatric Research Education and Clinical Center, Veterans Affairs Puget Sound Health Care System, Seattle, WA, USA; Oregon Health & Science University, Portland, OR, USA; Laboratory of Clinical and Experimental Endocrinology, Department of chronic diseases and aging, Katholieke Universiteit Leuven, Leuven, Belgium; Department of Endocrinology and Diabetes, Fiona Stanley Hospital, Perth, Western Australia, Australia; Medical School, University of Western Australia, Perth, Australia; Department of Internal Medicine and Clinical Nutrition, Institute of Medicine, Sahlgrenska Osteoporosis Centre, Centre for Bone and Arthritis Research at the Sahlgrenska Academy, University of Gothenburg, Gothenburg SE-413 45, Sweden; Region Västra Götaland, Department of Drug Treatment, Sahlgrenska University Hospital, Gothenburg, Sweden

**Keywords:** incident fractures, testosterone, SHBG, men

## Abstract

**Context:**

As men age, circulating testosterone (T) decreases, circulating SHBG increases, and the risk of fracture increases. It is unclear if circulating T, independently of comorbidities, is associated with fracture risk in men.

**Objectives:**

To determine associations for T and SHBG with incident fractures in men.

**Methods:**

We utilized the large (n = 205 973 participants, 11 088 any fracture cases, 1680 hip fracture cases, 1366 forearm fracture cases) and well-characterized UK Biobank cohort. Associations were modeled using Cox regressions, adjusting for multiple comorbidities/covariates, imputing for missing information, and assessing nonlinearity using cubic splines.

**Results:**

For T, not considering SHBG, there was a nonlinear association with hip but not forearm fractures, with the lowest risk in the second quintile. However, in models adjusted for SHBG or using calculated free T, lower T was associated with a higher risk for fractures at all evaluated bone sites. Lower SHBG was strongly associated with a lower risk of hip and forearm fractures (Q1 vs Q5, hip 0.55, 0.47-0.65; forearm 0.62, 0.52-0.74).

**Conclusion:**

Low circulating SHBG is strongly associated with a low risk of fracture at all evaluated bone sites, while the associations of circulating T with fracture risk are of lesser magnitude, nonlinear, inconsistent among fracture site, and affected by adjustment for SHBG. These findings demonstrate that circulating SHBG, rather than T, is a major independent biomarker of fracture risk in men. Consequently, both total T and SHBG should be assessed when examining the relationship of endogenous T concentrations with fractures in middle-aged to older men.

Osteoporotic fractures increase with age as bone mass decreases, bone microarchitecture deteriorates, and the propensity to fall increases. Although osteoporotic fractures are more common in women than in men, as many as 1 in 4 men sustain an osteoporotic fracture during their lifetime (1). Men have higher mortality than women following a fracture, thus it is of concern that men at high risk of fractures are often untreated (2, 3).

As men age, their circulating testosterone (T) decreases, circulating SHBG increases, and risk of fracture increases. However, it is unclear if circulating T per se, independently of comorbidities, contributes to the increased fracture incidence in older men. Prospective observational studies examining the associations between circulating T and incident fractures have yielded inconsistent results (4-13). These studies have included up to 4324 men with up to 342 incident fracture cases and have reported inconsistent associations of circulating T and incident fracture risk. This inconsistency may be due to the fact that the study populations may differ concerning the prevalence of hypogonadism, risk of falls, and muscle mass of the individuals, factors that could impact the association between T and fracture risk. In contrast, most previous prospective observational studies demonstrate that high circulating SHBG (4-10, 13, 14) and low circulating calculated free T (cFT) (4-6, 8, 9, 11, 14), calculated using total T and SHBG (15), are associated with increased fracture risk in men.

In men who are frankly hypogonadal, T treatment improves bone mineral density (BMD) (16-18). In older men, T treatment had no or minor effects on BMD in early short and small studies (19-22). However, the more recent Bone Trial of the Testosterone trials (T-Trials) showed that T treatment with a gel for 1 year increased volumetric BMD and estimated bone strength in older men with low T (23). Interestingly, the treatment-induced change in circulating estradiol, not the change in circulating T, was the best predictor of the change in volumetric BMD in that study (24). In the recent T for Bone (T4Bone) trial, a substudy of the T4DM trial, which included men ≥50 years of age with either impaired glucose tolerance or newly diagnosed type 2 diabetes (25), treated with injectable T undecanoate or placebo for 2 years, T treatment increased both volumetric BMD as analyzed by computed tomography and areal BMD as analyzed by dual energy x-ray absorptiometry (26). However, neither the T-Trials nor the T4Bone trial were powered to determine whether T treatment affects fracture risk. Unexpectedly, in the recent large placebo-controlled TRAVERSE trial (n = 5204), treating men with T less than 300 ng/dL with a high risk of cardiovascular disease with a T gel, T treatment increased the risk of clinical fractures, but the underlying mechanism is unknown [placebo: 64 fractures; testosterone: 91 fractures; hazard ratio (HR) for fractures 1.43; 95% confidence interval (CI), 1.04-1.97] (27). The fracture incidence also appeared to be higher for all other fracture end points in that study (27). In summary, many of the T treatment studies included men with different pathologies and risk factors that could influence the effect of T on bone.

Thus, the role of exogenous and endogenous T for fracture risk in men is unclear. The main aim of the present study was to determine associations of endogenous circulating T, SHBG, and T considering SHBG (either as cFT or T adjusted for SHBG) with incident fractures at different bone sites in men. To determine these associations, adjusting for multiple comorbidities/covariates and imputing for missing information in the models, we used the UK Biobank that constitutes the by far largest (n = 205 973 participants, 11 088 fracture cases) male prospective cohort (28).

## Materials and Methods

### The UK Biobank

From 2006 to 2010, the prospective UK Biobank cohort study recruited over 500 000 community-dwelling individuals aged 37 to 73 years from across the United Kingdom. Participants provided biological samples, completed questionnaires, underwent assessments, and were interviewed by nurses. Blood was collected for future analysis, and the self-reported interval between consumption of food and drink and blood sampling, ie, fasting time, was recorded. Follow-up using record linkage to all health service encounters and mortality data is ongoing. The UK Biobank has ethical approval from the Northwest Multicentre Research Ethics Committee (reference 11/NW/0382), and all participants provided informed consent (28). This research was conducted using the UK Biobank resource under application number 54 680.

### Variables of Interest

#### Exposures

Blood samples were collected throughout the day and analyzed in the UK Biobank core laboratory (29, 30). Serum total T was quantified using a competitive binding chemiluminescent immunoassay (DXI 800; Beckman Coulter Cat# 33560, RRID:AB_2905661, UK) with an analytical range of 0.35 to 55.5 nmol/L and coefficients of variation of 8.3% for low concentrations, 3.7% for medium concentrations, and 4.2% for high concentrations (29, 30). Serum SHBG was quantified using a 2-step sandwich chemiluminescent immunoassay (DXI 800; Beckman Coulter Cat# A48617, RRID:AB_2893035) with an analytical range of 0.33 to 242 nmol/L and coefficients of variation of 5.7% for low concentrations, 5.3% for medium concentrations, and 5.2% for high concentrations (29, 30). cFT was calculated using the Vermeulen method, using T, SHBG, and fixed albumin concentration (42 g/L) (15).

#### Fracture outcomes

Follow-up of incident events was from the baseline survey (March 2006 to October 2010) until the corresponding UK Biobank censoring date for each country (as of July 12, 2023; October 31, 2022 for England; August 31, 2022 for Scotland; May 31, 2022 for Wales) where the baseline assessment had taken place. Incident events during follow-up were identified using the International Classification of Diseases diagnosis codes from hospital admissions, listed in any position, for each of any fracture, hip fracture, or forearm fracture [Supplementary Table S1 (31)]. Follow-up times for participants who did not experience an incident event prior to being lost to follow-up, death, or end of follow-up were retained as censored observations.

#### Covariates

Participants’ age, body mass index, waist circumference, alcohol consumption, diet (red meat consumption: high vs low vs none), educational qualifications (completed university/college vs not), ethnicity (White vs not White), level of physical activity, living with partner (yes vs no), smoking status, use of medications (anticonvulsants, glucocorticoids, opioids, vitamin D supplementation, total number of medications taken), and comorbidities (chronic obstructive pulmonary disease, primary hyperparathyroidism, renal impairment, secondary osteoporosis, thyroid disease) were derived from data collected at the baseline assessment [Supplementary Methods (31)]. Total number of medications was included as a proxy for overall comorbidity status (32). The time of blood sample collection and vitamin D concentration in blood serum assayed using chemiluminescent immunoassay were also obtained. Secondary osteoporosis (comprised of men reporting type 1 diabetes, chronic liver disease, or osteogenesis imperfecta) and glucocorticoid use were identified using definitions provided in another UK Biobank study (33). Geographic regions were obtained by grouping baseline assessment centers into 1 of 10 broader spatial units (South West, South East, London, East Midlands, West Midlands, Yorkshire & The Humber, North East, North West, Scotland, Wales) (34).

### Statistical Analyses

The risk of each fracture outcome associated with each baseline sex hormone (T, SHBG, cFT) concentration was estimated using Cox proportional hazards modeling. Four models of increasing complexity were fitted, including sex hormone, time of blood sampling, geographic region, and participant age (model 1); model 1 terms plus thyroid disease, renal impairment, ethnicity, and other Fracture Risk Assessment Tool.(FRAX)-related covariates (body mass index, fracture in past 5 years, smoking status, glucocorticoid use, secondary osteoporosis, alcohol consumption) (model 2); model 2 terms plus living with partner, education, diet, physical activity, waist circumference, serum vitamin D, and comorbidity/medication usage (chronic obstructive pulmonary disease, opioids, anticonvulsants, vitamin D supplementation, number of medications) (model 3); model 3 terms plus T for analyses of SHBG or SHBG for analyses of T (model 4). Continuous variables were modeled using restricted cubic splines with outer knots values placed at the 5th and 95th percentiles and inner knots at the 27.5th, 50th, and 72.5th percentiles (35). Geographic region was modeled as a stratification factor to account for potential spatial variability in demographic, lifestyle, and health factors. Per-variable and global tests of the proportional hazards were conducted from the fit of each model to the first of the imputed datasets and Schoenfeld residual plots inspected (36). Covariates showing a departure from proportional hazards were analyzed as stratification factors instead of as model terms; see Supplementary Methods (31, 35).

Each model was fitted to 40 multiply imputed versions of the dataset after excluding men with pituitary disease, infertility, orchidectomy, congenital adrenal hyperplasia, missing baseline sex hormone concentration; men receiving androgen, antiandrogen, 5α-reductase inhibitors, or other hormone medications or osteoporosis therapies; men with a history of rheumatoid arthritis, malnutrition or malabsorption, vitamin D deficiency; or men who had withdrawn consent. Fully conditionally specified imputations were done including fracture outcome and all predictors from the full model (model 4 for T and SHBG and model 3 for cFT analyses). Cubic polynomial terms were constructed for variables that were modeled using restricted cubic splines to ensure that imputation models were congenial with the analysis models (37). Further details are provided in Supplementary Methods (31). Multiply imputed estimates were pooled using Rubin's rules (38).

Estimates of the median follow-up times were calculated using the reverse Kaplan–Meier method (39). Plots of the estimated HR and 95% CIs for each baseline sex hormone concentration, calculated relative to the median of the highest (fifth) sample quintile, were constructed using marginal predictions from each fitted model. HRs and 95% CIs were also tabulated for medians of sample quintiles.

Competing risk regression models were fitted to estimate the role of baseline hormone (testosterone, SHBG, cFT) concentration on the 10-year predicted risk of each fracture outcome, lowered by the occurrence of the competing risk of death. A Fine–Gray model including all model terms plus the country of the assessment center (England, Scotland, Wales) was fitted to each of the fracture Multiple Imputation using Chained Equations-imputed datasets in R using the fastcmprsk package with the maximum number of iterations set to 10 000. Marginal predictions of cumulative incidence at 10 years were then predicted from each fitted model for this cohort of UK Biobank men. Analyses were conducted using R version 4.3.1 (40).

## Results

### Study Cohort

After excluding participants who had withdrawn consent, there were data available for 229 066 men. Excluding men who were taking androgen, antiandrogen, 5α-reductase inhibitors, or other hormone medications and men with pituitary disease, infertility, orchidectomy, or congenital adrenal hyperplasia left 224 211. Additional exclusions of men on osteoporosis therapies, with a history of rheumatoid arthritis, malnutrition, malabsorption, or vitamin D deficiency left 221 597 [Supplementary Fig. S1 (31)]. Further exclusions due to missing sex hormone measurements at baseline left n = 205 973 for T analyses, n = 190 607 for SHBG analyses, and n = 189 585 for cFT analyses [cFT estimation requires both T and SHBG, Supplementary Fig. S1 (31)].

### Participant Characteristics

The analysis cohort comprised men who were middle- to older-aged at baseline, with ages ranging from 37 to 73 years (median = 58 years). The duration of follow-up (median with interquartile range) was 13.6 years (12.9 to 14.3 years) for any fracture. During follow-up, 11 088 men (5.0%) recorded a fracture at any bone site [1680 hip fracture cases and 1366 forearm fracture cases (Supplementary Table S2 (31)]. Those who experienced a hip fracture during follow-up were slightly older (median age = 63 years) than those who experienced a forearm fracture (median age = 57 years; Table 1). The majority of men were not obese, vitamin D sufficient, of White ethnicity, living with a partner, drinking no or moderate alcohol, consuming a low red meat diet (beef, lamb, or pork intake 2-4 times per week or less), performing sufficient or additional physical activity, never or previous smokers, and relatively healthy, taking 0 to 2 regular prescription medications. Median concentrations in blood were 11.6 nmol/L for T and 36.9 nmol/L for SHBG for all participants but higher for those who experienced subsequent fracture, although the differences for T were relatively small [Supplementary Table S2 (31)].

**Table 1.** Hazard ratios of different types of fracture event by quintiles of T*^a^*

	Model	Q1 (lowest T)	Q2	Q3	Q4	Q5 (highest T)
	**Median T (nmol/L)**	7.70	9.89	11.61	13.53	16.69
	**Median T (ng/dL)**	222	285	335	390	481
		**n = 41 197**	**n = 41 201**	**n = 41 191**	**n = 41 191**	**n = 41 193**
Any fracture: 11,008 events						
		2230 events	2107 events	2099 events	2193 events	***2379 events***
Models without SHBG	Model 1	0.92 (0.88-0.97)	0.84 (0.80-0.88)	0.90 (0.84-0.95)	0.93 (0.90-0.96)	ref.
	Model 2	0.96 (0.91-1.02)	0.89 (0.84-0.94)	0.94 (0.89-1.00)	0.96 (0.93-0.99)	ref.
	Model 3	0.93 (0.88-0.98)	0.89 (0.84-0.94)	0.94 (0.89-1.00)	0.97 (0.94-1.00)	ref.
Model with SHBG	Model 4	1.24 (1.16-1.33)	1.12 (1.05-1.19)	1.11 (1.04-1.18)	1.06 (1.02-1.10)	ref.
Hip fracture: 1,680 events						
		343 events	278 events	301 events	361 events	***397 events***
Models without SHBG	Model 1	0.72 (0.63-0.82)	0.65 (0.57-0.74)	0.74 (0.64-0.86)	0.86 (0.79-0.93)	ref.
	Model 2	0.90 (0.78-1.03)	0.80 (0.70-0.92)	0.87 (0.74-1.01)	0.94 (0.86-1.02)	ref.
	Model 3	0.79 (0.69-0.91)	0.76 (0.66-0.87)	0.84 (0.72-0.98)	0.93 (0.86-1.01)	ref.
Model with SHBG	Model 4	1.28 (1.08-1.51)	1.13 (0.97-1.33)	1.09 (0.93-1.29)	1.07 (0.98-1.17)	ref.
Forearm fracture: 1,366 events						
		255 events	289 events	273 events	253 events	***296 events***
Models without SHBG	Model 1	0.97 (0.84-1.12)	0.92 (0.79-1.06)	0.96 (0.81-1.13)	0.95 (0.87-1.04)	ref.
	Model 2	1.02 (0.88-1.19)	0.97 (0.83-1.13)	1.00 (0.84-1.18)	0.97 (0.89-1.07)	ref.
	Model 3	1.00 (0.86-1.17)	0.97 (0.83-1.13)	1.00 (0.84-1.19)	0.98 (0.89-1.07)	ref.
Model with SHBG	Model 4	1.51 (1.25-1.82)	1.35 (1.13-1.61)	1.25 (1.04-1.51)	1.11 (1.00-1.22)	ref.

^*a*^Pooled estimates from multiple imputations. Hazard ratios calculated for the medians of testosterone within each sample quintile (Q1-Q5), relative to the median for Q5. Quintile boundaries were Q1/2 8.93 nmol/L (257 ng/dL), Q2/3 10.76 nmol/L (310 ng/dL), Q3/4 12.50 nmol/L (360 ng/dL), and Q4/5 14.78 nmol/L (426 ng/dL).

Model 1 included terms for testosterone, age, and time of blood sampling, with UK region modeled as a stratification factor (see Methods).

Model 2 included model 1 terms + ethnicity (White vs not White), alcohol consumption, smoking status, body mass index, use of glucocorticoids, fracture in past 5 years, renal impairment, secondary osteoporosis, and thyroid disease.

Model 3 included model 2 terms + educational attainment; living with partner; diet (red meat: high vs low vs none); physical activity; waist circumference; chronic obstructive pulmonary disease; and use of anticonvulsants, opioids, and vitamin D supplements, with the number of medications included as a proxy for overall comorbidity status.

Model 4 included model 3 terms + SHBG.

Abbreviation: T, testosterone.

### Testosterone Associations

#### T analyses not considering SHBG

**Figure 1.**
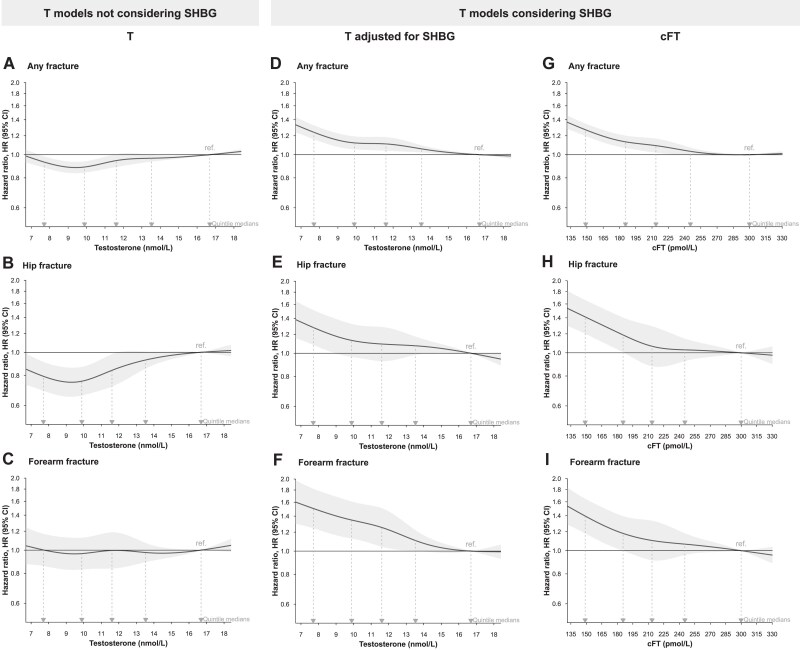
Estimated association of baseline serum testosterone concentration with risk of fracture in models with or without consideration of SHBG. (A–C) Estimates for testosterone models not considering SHBG, model 3, adjusted for time of blood sampling; geographic region; thyroid disease; renal impairment; ethnicity (White vs not White); participant age; other FRAX-related clinical risk factors: body mass index, fracture in past 5 years, smoking status, glucocorticoid use, secondary osteoporosis, and alcohol consumption; living with partner status; educational attainment; diet (red meat: high vs low vs none); physical activity; waist circumference; chronic obstructive pulmonary disease; serum vitamin D concentration; opioids; anticonvulsants; vitamin D supplementation; and total number of medications (proxy for overall comorbidity status). (D–I) Estimates for testosterone models considering SHBG either by (D–F) adjusting for SHBG and model 3 covariates (ie, model 4) or (G–I) using calculated free testosterone as exposure and model 3 covariates. Shaded areas are 95% confidence intervals and the locations of hazard ratios (medians of sample quintiles, as presented in Table 1 for testosterone and Table 2 for calculated free testosterone) are indicated. Horizontal axes are truncated to exclude values outside of boundary knots, where data are sparsely distributed and trends are constrained to linearity. Abbreviation: FRAX, Fracture Risk Assessment Tool.

The estimated HRs of any and hip fractures from model 1, which controlled for baseline age, time of blood sampling, and region, demonstrated nonlinear associations with baseline T, being lower (that is, HR < 1) relative to the reference value at the median of the fifth quintile (Q5; 16.7 nmol/L) and lowest near the median of the second quintile [Q2, 9.9 nmol/L; Supplementary Fig. S2 (31)]. These nonlinear associations remained but were slightly attenuated after adjustments for FRAX-related clinical risk factors [model 2, Supplementary Fig. S2 (31)] and FRAX + comorbidity-related predictors (model 3, Fig. 1). There were no statistically significant associations for T with risk of forearm fractures [Fig. 1, Supplementary Fig. S2 (31)]. When comparing quintiles of T using the model adjusted for FRAX- and comorbidities-related predictors (model 3), there was a nonlinear association of T with hip but not forearm fractures, with the lowest risk in the second quintile (Q2, HR, 95% CIs Q2 vs Q5 hip fracture 0.76, 0.66-0.87; forearm fracture 0.97, 0.83-1.13; Table 1).

#### T analyses adjusting for SHBG

For fractures at all evaluated bone sites, subsequent adjustment for baseline SHBG (model 4) resulted in elevated estimates of fracture risk (that is, HR > 1) for men with T concentrations lower than the Q5 median, with the highest risk for the men with the lowest T concentrations (Fig. 1; Table 1).

#### Analyses using cFT

Using cFT, lower cFT was associated with a higher risk for fractures at all evaluated bone sites [Fig. 1, Supplementary Fig. S3 (31), Table 2]. It is noteworthy that the estimated associations for cFT (any fracture HR 1.24, 1.16-1.33, Q1 vs Q5) with risk of fracture at different bone sites resemble those for testosterone adjusted for SHBG (any fracture HR 1.27, 1.20-1.35, Q1 vs Q5, Fig. 1).

**Table 2.** Hazard ratios of different types of incident fractures by quintiles of cFT (pmol/L)*^a^*

Model	Q1	Q2	Q3	Q4	Q5
**Median cFT (pmol/L)**	**148.9**	**185.6**	**213.6**	**245.4**	**299.9**
**Median cFT (pg/mL)**	**42.9**	**53.5**	**61.6**	**70.8**	**86.5**
	**n = 37 925**	**n = 37 920**	**n = 37 906**	**n = 37 922**	**n = 37 912**
Any fracture: 10,123 events					
	2591 events	2135 events	1867 events	1848 events	1682 events
Model 1	1.38 (1.30-1.46)	1.16 (1.10-1.24)	1.11 (1.04-1.18)	1.04 (1.00-1.08)	ref.
Model 2	1.31 (1.24-1.39)	1.14 (1.07-1.21)	1.09 (1.02-1.16)	1.03 (0.99-1.07)	ref.
Model 3	1.27 (1.20-1.35)	1.13 (1.07-1.20)	1.09 (1.02-1.17)	1.03 (1.00-1.07)	ref.
Hip fracture: 1,536 events					
	524 events	342 events	265 events	237 events	168 events
Model 1	1.62 (1.38-1.90)	1.25 (1.06-1.48)	1.11 (0.92-1.34)	1.05 (0.94-1.17)	ref.
Model 2	1.51 (1.29-1.77)	1.21 (1.02-1.43)	1.08 (0.89-1.31)	1.03 (0.93-1.15)	ref.
Model 3	1.41 (1.20-1.66)	1.19 (1.00-1.40)	1.07 (0.88-1.29)	1.03 (0.92-1.15)	ref.
Forearm fracture: 1,249 events					
	304 events	248 events	234 events	236 events	227 events
Model 1	1.46 (1.24-1.72)	1.20 (1.02-1.41)	1.12 (0.92-1.35)	1.07 (0.97-1.19)	ref.
Model 2	1.42 (1.21-1.68)	1.18 (1.00-1.40)	1.10 (0.91-1.33)	1.06 (0.96-1.18)	ref.
Model 3	1.40 (1.18-1.65)	1.18 (1.00-1.39)	1.10 (0.91-1.33)	1.06 (0.96-1.18)	ref.

^*a*^Pooled estimates from multiple imputation. Hazard ratios calculated for the medians of cFT within each sample quintile (Q1-Q5), relative to the median for Q5.

Quintile boundaries were Q1/2 169.7 pmol/L, Q2/3 199.6 pmol/L, Q3/4 228.4 pmol/L, Q4/5 266.5 pmol/L.

Model 1 included terms for cFT, age, and time of blood sampling, with UK region modeled as a stratification factor (see Methods).

Model 2 included model 1 terms + ethnicity (White vs not White), alcohol consumption, smoking status, body mass index, use of glucocorticoids, fracture in past 5 years, renal impairment, secondary osteoporosis, and thyroid disease.

Model 3 included model 2 terms + educational attainment; living with partner; diet (red meat: high vs low vs none); physical activity; waist circumference; chronic obstructive pulmonary disease; and use of anticonvulsants, opioids, and vitamin D supplements, with the number of medications included as a proxy for overall comorbidity status.

Abbreviation: cFT, tree testosterone.

#### SHBG associations

**Figure 2.**
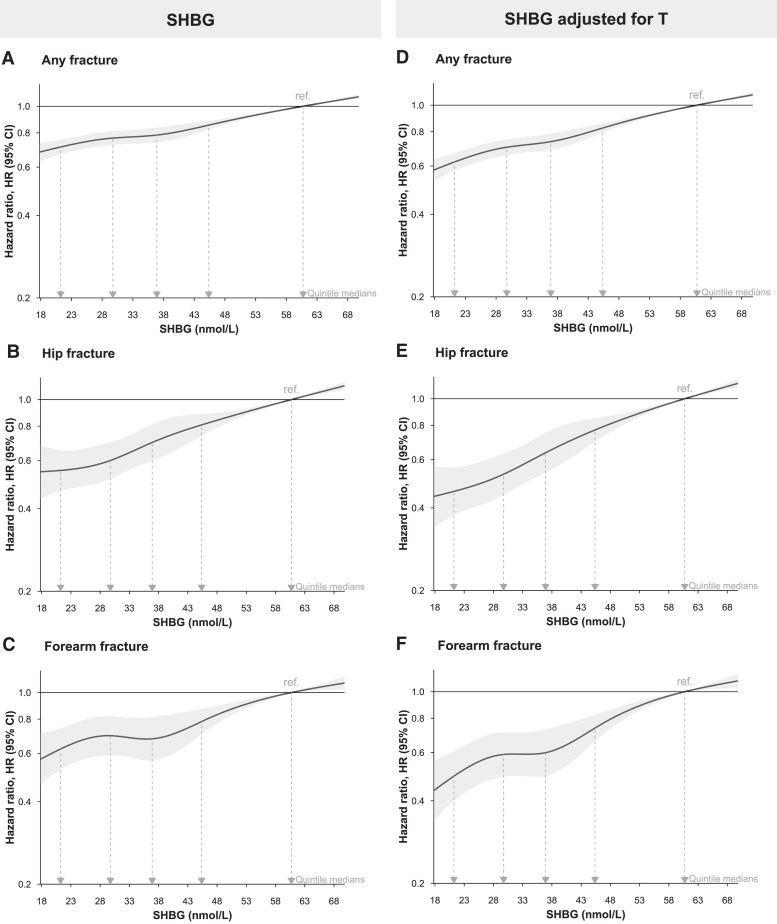
Estimated association of baseline serum SHBG concentration with risk of fracture. (A–C) Estimates for SHBG using model 3, adjusted for time of blood sampling; geographic region; thyroid disease; renal impairment; ethnicity (White vs not White); participant age; other FRAX-related clinical risk factors: body mass index, fracture in past five years, smoking status, glucocorticoid use, secondary osteoporosis, and alcohol consumption; living with partner status; educational attainment; diet (red meat: high vs low vs none); physical activity; waist circumference; chronic obstructive pulmonary disease; serum vitamin D concentration; opioids; anticonvulsants; vitamin D supplementation; and total number of medications (proxy for overall comorbidity status). (D–F) Estimates for SHBG models adjusting for testosterone and model 3 covariates (ie, model 4). Shaded areas are 95% confidence intervals and the locations of hazard ratios (medians of sample quintiles, as presented in Table 3) are indicated. Horizontal axes are truncated to exclude values outside of boundary knots, where data are sparsely distributed and trends are constrained to linearity. Abbreviation: FRAX, Fracture Risk Assessment Tool.

In the base model (model 1), lower SHBG concentrations were associated with a lower risk of fractures at all evaluated bone sites [Supplementary Fig. S4 (31)]. Further adjustments for FRAX-related predictors [model 2, Supplementary Fig. S4 (31)] and FRAX + comorbidity-related predictors (model 3, Fig. 2) did not result in substantively different estimates. When comparing quintiles of SHBG using the model adjusted for FRAX- and comorbidity-related predictors (model 3), lower SHBG concentrations were strongly associated with lower risk of any, hip, and forearm fractures (HR Q1 vs Q5, any fracture 0.71, 0.67-0.75, hip fracture 0.55, 0.47-0.65; forearm fracture 0.62, 0.52-0.74, Table 3). This was reflected by a higher absolute 10-year risk, taking the competing risk of death into account, for any, hip, and forearm fractures for men in quintile 5 (2.88%, 0.33%, and 0.47%, respectively) compared with men in quintile 1 (2.10%, 0.18%, and 0.30%, respectively) of SHBG [Supplementary Table S3 (31)]. Subsequent adjustment for baseline T (Fig. 2) did not substantively impact trends, except for estimating slightly lower HRs for the median of Q1 relative to that of Q5 (Fig 2 and Table 3).

**Table 3.** Hazard ratios of different types of incident fractures by quintiles of SHBG (nmol/L)*^a^*

	Model	Q1	Q2	Q3	Q4	Q5
	**Median SHBG (nmol/L)**	**21.2**	**29.7**	**36.9**	**45.4**	60.7
		**n = 38 161**	**n = 38 116**	**n = 38 091**	**n = 38 136**	**n = 38 103**
Any fracture: 10,193 events						
		1656 events	1783 events	1901 events	2099 events	***2754 events***
Models without T	Model 1	0.69 (0.65-0.73)	0.73 (0.69-0.77)	0.76 (0.71-0.81)	0.82 (0.79-0.85)	ref.
	Model 2	0.72 (0.68-0.77)	0.77 (0.72-0.81)	0.79 (0.74-0.84)	0.85 (0.82-0.88)	ref.
	Model 3	0.71 (0.67-0.75)	0.77 (0.72-0.81)	0.79 (0.74-0.84)	0.85 (0.82-0.89)	ref.
Model with T	Model 4	0.62 (0.58-0.67)	0.70 (0.66-0.75)	0.74 (0.69-0.79)	0.83 (0.79-0.86)	ref.
Hip fracture: 1,546 events						
		144 events	212 events	268 events	338 events	***584 events***
Models without T	Model 1	0.48 (0.41-0.56)	0.52 (0.45-0.60)	0.62 (0.53-0.71)	0.73 (0.67-0.80)	ref.
	Model 2	0.61 (0.51-0.72)	0.64 (0.55-0.74)	0.73 (0.63-0.85)	0.82 (0.75-0.90)	ref.
	Model 3	0.55 (0.47-0.65)	0.60 (0.51-0.70)	0.70 (0.60-0.81)	0.81 (0.74-0.89)	ref.
Models with T	Model 4	0.46 (0.38-0.56)	0.53 (0.45-0.63)	0.64 (0.54-0.75)	0.77 (0.70-0.85)	ref.
Forearm fracture: 1,255 events						
		219 events	220 events	236 events	239 events	***341 events***
Models without T	Model 1	0.62 (0.53-0.72)	0.68 (0.58-0.80)	0.67 (0.56-0.80)	0.77 (0.69-0.85)	ref.
	Model 2	0.63 (0.53-0.75)	0.70 (0.59-0.82)	0.68 (0.57-0.82)	0.78 (0.70-0.87)	ref.
	Model 3	0.62 (0.52-0.74)	0.70 (0.59-0.82)	0.68 (0.57-0.81)	0.78 (0.71-0.87)	ref.
Models with T	Model 4	0.49 (0.40-0.61)	0.59 (0.49-0.71)	0.60 (0.49-0.73)	0.74 (0.66-0.82)	ref.

^*a*^Pooled estimates from multiple imputation. Hazard ratios calculated for the medians of SHBG within each sample quintile (Q1-Q5), relative to the median for Q5.

Quintile boundaries were Q1/2 25.9 nmol/L, Q2/3 33.3 nmol/L, Q3/4 40.8 nmol/L, Q4/5 51.3 nmol/L.

Model 1 included terms for SHBG, age, and time of blood sampling, with UK region modeled as a stratification factor (see Methods).

Model 2 included model 1 terms + ethnicity (White vs not White), alcohol consumption, smoking status, body mass index, use of glucocorticoids, fracture in past 5 years, renal impairment, secondary osteoporosis, and thyroid disease.

Model 3 included model 2 terms + educational attainment; living with partner; diet (red meat: high vs low vs none); physical activity; waist circumference; chronic obstructive pulmonary disease; and use of anticonvulsants, opioids, and vitamin D supplements, with the number of medications included as a proxy for overall comorbidity status.

Model 4 included model 3 terms + T.

Abbreviation: T, testosterone.

## Discussion

The role of both exogenous and endogenous T for fracture risk in men is unclear. Conducting analyses in the large UK Biobank prospective cohort study, we found modest, nonlinear associations between circulating T with any fractures and incident hip fractures but not forearm fractures, with the lowest risk near the median of the second quintile. However, additional adjustment for SHBG revealed inverse associations of circulating T with the risk of fractures at all 3 bone sites. By contrast, lower SHBG was strongly associated with a lower risk of fractures at all investigated bone sites, and additional adjustment for T did not alter the results. These findings demonstrate that circulating SHBG, rather than T, is a major independent biomarker of fracture risk in men.

Previous prospective studies examining the associations between circulating T concentrations and incident fractures have yielded inconsistent results (4-13), possibly due to smaller sample sizes and differences in study populations. Compared with the largest of these studies, the present study includes over 30 times more fracture cases, owing to the large size and relatively long follow-up time of the UK Biobank (4-13). The large number of fracture cases enabled us to thoroughly evaluate possible nonlinear relationships between circulating T and incident fractures at different bone sites separately. We observed that circulating T was associated with fractures at any bone site in a nonlinear manner and that this association remained after adjustments for known clinical risk factors for fractures and comorbidities. Interestingly, this nonlinear association was bone-site specific and observed for hip fractures but not forearm fractures. When comparing quintiles of T, the lowest hip fracture risk was observed for men in the second quintile, with a gradual increase of hip fracture risk at higher quintiles and the highest risk observed for men in quintile 5. A possible explanation for the relatively high risk at the lowest quintile could be that these men are unhealthier than those with higher T levels and that, despite our efforts to correct for multiple confounding factors, there is residual confounding.

Similar to the current study, a nonlinear association was observed for T and fracture risk in the Health In Men Study with the highest risk for the lowest and highest quintiles of T (13). Thus, the present large study establishes that higher circulating T per se, not considering SHBG and within the normal range (starting from Q2, the lowest quartile), is associated with higher fracture risk in middle-aged to older men. This observational finding is in line with the recent unexpected finding from the TRAVERSE trial showing that T treatment of men, enhancing circulating T levels within the physiological range, increased the risk of fractures compared with placebo (27). However, the underlying mechanism for this finding is unknown and most likely not caused by reduced BMD as the T-Trials and the T4Bone studies showed that T treatment of men increased different BMD parameters and improved bone microarchitecture compared with placebo (23, 26). Future studies should determine the effect of T treatment on risk of falls and other factors influencing the fracture risk. Given the unexpected result that the fracture incidence was higher among men who received T than among those who received placebo in the TRAVERSE trial (27), it was recently speculated that T treatment may promote an active lifestyle, subsequently associated with a higher risk of trauma-induced fractures (41).

In the present study, we observed that low circulating SHBG was strongly associated with a low risk of fractures at all evaluated bone sites, and this association remained after adjustment for circulating T. The association between SHBG and fracture risk is in line with previous findings from smaller observational studies (4-10, 13, 14). In contrast, we recently observed that low SHBG was associated with an increased risk of myocardial infarction in men in the UK Biobank (42), demonstrating that high SHBG is not simply a marker of general poor health in these men. A role of SHBG for fracture risk is supported by recent 2-sample Mendelian randomization (MR) studies showing that higher genetically predicted circulating SHBG is causally associated with higher risk of fractures (43, 44) in sex-combined analyses. However, there is no sex-stratified MR study showing a causal effect of circulating SHBG on fracture risk in men. SHBG is mainly known to bind sex hormones and thereby influence their transport from the circulation into peripheral sex steroid target tissues, a concept supported by affected intratissue sex steroid levels in SHBG transgenic mice (45). Part of the substantial role of high SHBG on fracture risk may be mediated not only by modulating the effect of testosterone but also of estradiol, a known regulator of BMD and fracture risk in men, as demonstrated using MR (46, 47). However, the possibility that SHBG may have effects on its own cannot be excluded. Some studies have suggested that SHBG may bind to a receptor, and following steroid binding to SHBG, the receptor activates adenylate cyclase; however, the identity of the receptor is not known (48). Further studies are warranted to determine the mechanism for SHBG to affect fracture risk.

When considering SHBG in the models evaluating the association between circulating T and fracture risk, either by adjusting for SHBG or using SHBG in the calculations of cFT, the associations between T and fracture risk changed direction and were strengthened. In models considering SHBG, circulating T was linearly, but inversely, associated with fracture risk, contrasting with the direct linear associations of SHBG with incident fractures. These results suggest that the observed associations of T with fracture risk were heavily influenced or driven by SHBG. In contrast, the strong associations between SHBG and fracture risk were largely unaffected by adjustment for T. Based on the findings in the present study, we propose that not only total T but also SHBG concentrations should be assessed when examining the relationship of sex steroids and bone health in men. The additional value of the SHBG measurement may be due to SHBG's capacity to modulate the effect of not only circulating T (partly captured by cFT) but also estradiol on bone health. Taken together, these findings suggest that SHBG, rather than T, is a strong independent biomarker of fracture risk in men. This was further illustrated by substantially higher absolute 10-year risk for hip fractures for men in quintile 5 compared with men in quintile 1 of SHBG, suggesting that high levels of SHBG should be considered as a clinical risk factor for fractures in men.

The current study has several strengths, including the large size, long follow-up, detailed characterization of the participants, and high number of fractures of the UK Biobank that allowed us to analyze fractures at different bone sites separately, determine possible nonlinear associations, and adjust for many relevant comorbidities/covariates. In addition, we imputed for missing information that enabled us to adjust for multiple comorbidities/covariates without reducing the number of participants included in the different models used.

The current study also has several limitations. Although we used many covariates, we cannot exclude residual confounding due to missing covariates. As it is well known that sex steroids are associated with BMD and falls in men (46, 49), it is a limitation of the present study that additional models also adjusting for BMD and falls were not added. Another limitation is that the study is purely observational. Future sex-stratified MR studies would be important to determine the possible causal role for SHBG and T on fracture risk in men. In the UK Biobank, sex steroids were quantified using immunoassays instead of state-of-the art mass spectrometry. Although these methodologies correlate rather well, the absolute level of T differs (50); we, therefore, presented data as continuous variables and in quintiles to avoid using specific thresholds of T obtained by immunoassay. It should be emphasized that it is a limitation of the present study that valid estradiol measurements were not available in the UK Biobank, with 92% of the estradiol levels given below the detection limit of the immunoassay (51). In addition, serum dihydrotestosterone was not analyzed in the UK Biobank cohort. Considering the important links between estradiol and bone health in men (14, 46, 47), as well as the capacity of SHBG to also bind to estradiol, it is likely that the association between high circulating SHBG and increased fracture risk may involve reduced bioavailability of not only T but also estradiol. Further large-scale studies with reliable estradiol measurements are required to determine to what extent the association between SHBG and fracture risk is mediated via estradiol bioavailability. Generally, only unbound steroids are considered to convey steroid actions, but since free T was not directly measured, we calculated the free levels of T using the commonly used method by Vermeulen (15), and there is controversy about the accuracy of those estimates. An additional limitation is the lack of a certified standard or quality control for the calculated free testosterone. Since the UK Biobank consists of mainly European participants who have a relatively narrow age range (37-73 years old), the results may not be representative for non-European populations or for populations of elderly individuals with the highest risk of fracture. In addition, the response rate for the UK Biobank was low, and participants in the UK Biobank may be healthier than the general population of the United Kingdom (52).

In conclusion, our results show that low circulating SHBG is strongly associated with a low risk of fractures in men. The associations for circulating T with fracture risk were weaker, nonlinear, and observed for hip but not forearm fractures. Importantly, the associations of T with fracture risk changed direction and were strengthened in models considering SHBG (via SHBG adjustment or use of cFT), whereas the associations of SHBG were robust to additional adjustment for T. These findings demonstrate that circulating SHBG, rather than T, is a major independent biomarker of fracture risk in men. Consequently, not only total T but also SHBG concentrations should be assessed when examining the relationship of endogenous T concentrations with health outcomes influenced by androgen-sensitive tissues in middle-aged to older men.

## Data Availability

Study protocol: Not applicable. Statistical code: May be made available on reasonable request (e-mail, bu.yeap@uwa.edu.au). Data set: Data from the UK Biobank are accessible to researchers via application to the UK Biobank (www.ukbiobank.ac.uk).
